# Supplementary material for: Double-Edged Sword Effect of Pyroptosis: The Role of Caspase-1/-4/-5/-11 in Different Levels of Apical Periodontitis
Source: Biomolecules. 2022 Nov 8;12(11):1660. doi: 10.3390/biom12111660 (PMC9687662; doi:10.3390/biom12111660)
Supplement: Supplementary file 1 [file biomolecules-12-01660-s001.zip › biomolecules-1924891 - Supplementary 1 - proofed.pdf]

biomolecules

MDPI

Article

Double-Edged Sword Effect of Pyroptosis: The Role of Caspase-1/-4/-5/-11 in Different Levels of Apical Periodontitis

Zhiwu Wu †, Mingming Li †, Xiaolin Ren, Rui Zhang, Jinfeng He, Li Cheng, Ran Cheng * and Tao Hu *

State Key Laboratory of Oral Diseases, Frontier Innovation Center for Dental Medicine Plus, National Clinical Research Center for Oral Diseases, Department of Preventive Dentistry, West China Hospital of Stomatology, Sichuan University, Chengdu 610041, China

* Correspondence: chengran@scu.edu.cn (R.C.); hutao@scu.edu.cn (T.H.); Tel.: +86-28-8550-3486(R.C. and T.H)

† These authors contributed equally to this work.

Citation: Wu, Z.; Li, M.; Ren, X.; Zhang, R.; He, J.; Cheng, L.; Cheng, R.; Hu, T. Double-Edged Sword Effect of Pyroptosis: The Role of Caspase-1/-4/-5/-11 in Different Levels of Apical Periodontitis. *Biomolecules* **2022**, *12*, 1660. https://doi.org/10.3390/biom12111660

Academic Editor(s): Masaru Yamaguchi

Received: 3 September 2022
Accepted: 3 November 2022
Published: date

Publisher's Note: MDPI stays neutral with regard to jurisdictional claims in published maps and institutional affiliations.

Copyright: © 2022 by the authors. Submitted for possible open access publication under the terms and conditions of the Creative Commons Attribution (CC BY) license (https://creativecommons.org/licenses/by/4.0/).

Materials and Methods

Clinical specimens

The study procedure was reviewed and approved by the Institutional Ethics Committee of West China Hospital of Stomatology (WCHSIRB-D-2020-324). Each subject signed a written consent form. Human apical periodontitis tissues (HAPT, a total number of 23) from healthy human donors (aged 18 to 64 years) were obtained from extracted human teeth or teeth that underwent periapical surgery at West China Hospital of Stomatology (Sichuan University, Chengdu, Sichuan, China). The inclusion criteria were that teeth were diagnosed as chronic AP with periapical radiolucent lesions and required periapical surgery or extraction. The exclusion criteria were patients with malignant tumours or autoimmune diseases and patients who smoked or had antibiotic use for at least 3 months. In addition, human periodontal ligaments (HPDL, a total number of 47) were collected from healthy non-smoking donors (aged 14 to 46 years) after premolar extraction for orthodontic reasons and third molar extraction.

Label-free proteomics

Samples were digested, and proteins were extracted in SDT buffer (4% SDS, 100 mM Tris-HCl, 1 mM DTT, pH 7.6). The amount of protein was quantified with the BCA Protein Assay Kit (Bio-Rad, Hercules, CA, USA). Protein digestion by trypsin was performed according to the filter-aided sample preparation (FASP) procedure described by Matthias Mann. The digest peptides of each sample were desalted on C18 Cartridges (Empore™ SPE Cartridges C18 (standard density), bed I.D. 7 mm, Volume 3 ml, Sigma-Aldrich, St Louis, MO, USA), concentrated by vacuum centrifugation and reconstituted in 40 µl of 0.1% (v/v) formic acid. The proteins were separated on a 12.5% SDS-PAGE gel. Protein bands were visualized by Coomassie Blue R-250 staining. LC–MS/MS analysis was performed on a Q Exactive mass spectrometer (Thermo Fisher Scientific, Waltham, MA, USA) that was coupled to Easy nLC (Proxeon Biosystems, Thermo Fisher Scientific) for 60/120/240 min. The MS raw data for each sample were combined and searched using MaxQuant 1.5.3.17 software for identification and quantitation analysis. Ultimately, bioinformatic analysis was performed.

Establishment of experimental AP (EAP) rat model

The procedures were approved by the Institutional Ethics Committee of West China Hospital of Stomatology (WCHSIRB-D-2020-387). All animal experiments were performed in accordance with the Chinese State Key Laboratory of Oral Diseases guidelines for animal welfare (NO·SCXK (111) 2009-09).

Female Sprague-Dawley (SD) rats, aged 12-16 weeks and weighting 220g, were purchased from Beijing Vital River Laboratory Animal Technology Co., Ltd. SPF (Specified

Pathogen Free) animals were housed 5 rats per cage in room on a 12:12 light/dark cycle with temperature of 22℃. The pulp of bilateral upper and lower first molar was exposed to oral environment using a #1/4 dental round bur as previously described [1]. The rats were randomly divided into five groups (n=7-8): 1) Group 1: no treatment. 2) Group 2, 1week after treatment.3) Group 3, 2 weeks after treatment. 4) Group 4, 3 weeks after treatment. Group 5, 4 weeks after treatment. Rats were weighed once a week and then their physical appearance was routinely recorded. Rats of each group were executed using cervical dislocation after being anesthetized with isoflurane after the abovementioned weeks. Mandibles and maxilla were collected until free of soft tissues, fixed with 4% paraformaldehyde at 4 °C for 72 h. Next, the mandibles were stored in 75% ethanol prior to micro-computed tomography (micro-CT) scan. Then the specimens were rinsed and decalcified with 10% ethylene diamine tetraacetic acid for 12 weeks, dehydrated, and embedded in paraffin. Serial sections of 5 μm thickness were cut in the mesiodistal direction for further experiments.

For the inhibition experiment using caspase-1 inhibitor VX765, Caspase-11 inhibitor Wedelolactone (Wed), female SD rats aged 2–3 months, weighing approximately 200 g were used to establish AP rat model. One week after treatment (caspase-1/11 was activated in apical region at 1week), VX765 [1] (50 mg/kg; Biochempartner, Hangzhou, Zhejiang, China) and Wed [2] (4 mg/kg; Biochempartner, Hangzhou, Zhejiang, China) dissolved in 20% cremophor (Sigma-Aldrich, St Louis, MO, USA) together were intraperitoneally injected once a day for 14 consecutive days [3]. The rats were randomly divided into three groups: AP+VX765+Wed group, n=6 rats×2; control rats received the corresponding vehicle without VX765 or Wed (AP group, n = 5 rats × 2). Rats were weighed once a week and then their physical appearance was routinely recorded. All rats were anesthetized at 4 weeks after pulp exposure and the mandibles and maxilla were fixed with 4% paraformaldehyde at 4 °C for 72 h. Next, the mandibles and maxilla were stored in 75% ethanol prior to micro-computed tomography (micro-CT) scan. Then the specimens were rinsed and decalcified with 10% ethylene diamine tetraacetic acid for 12 weeks, dehydrated, and embedded in paraffin. Serial sections of 5 μm thickness were cut in the mesiodistal direction for further experiments. The researchers were blinded to the group analyzed.

Micro-CT analysis

The mandibles and maxillary were placed in an airtight cylindrical sample holder. A Scanco μCT50 imaging system (Scanco Medical AG, Bassersdorf, Zurich, Switzerland) was used to assess the periapical bone loss of the distal root of the first mandibular molar and the disto-buccal root of the first maxillary molar. The scanning was performed at 70 kVp and 200 μA, with a resolution of 10 μm and 300-millisecond exposure time. The segment selection method was performed as previously described [1]. Bone volume relative to the total volume (BV/TV), trabecular number (Tb. N), trabecular thickness (Tb. Th) and trabecular space (Tb. Sp) were counted for quantification to evaluate bone loss of periapical regions [4].

Hematoxylin-eosin staining (HE), immunofluorescence and immunohistochemistry (IHC)

The slice of EAP that exhibited a clear root canal apex and clinical specimens were selected for staining. HE was performed according to the standard procedure (Biosharp Life Science, Hefei, Anhui, China).

Immunofluorescence dual labelling staining was used to assess the spatial distribution and localization of caspase-1, caspase-4/-5/-11 and CD68 in the HAPT and EAP. The immunoreaction sequences were as follows: 1) Primary antibody incubation: mouse anti-caspase-1 (sc-398715, Santa Cruz Biotechnology Inc. Dallas, CA, USA) and rabbit anti-caspase-4 (ab25898, Abcam, Cambridge, UK); mouse anti-caspase-1 (sc-398715, Santa Cruz Biotechnology) and rabbit anti-caspase-5 (ab40887, Abcam); rabbit anti-caspase-4

(ab25898, Abcam) and mouse anti-caspase-5 (sc-393346, Santa Cruz Biotechnology); rabbit anti-caspase-1 (ab1872, Abcam) and mouse anti-caspase-11 (sc-374615, Santa Cruz Biotechnology), mouse anti-CD68 (ab201340, Abcam) and rabbit anti-caspase1 (ab1872, Abcam), rabbit caspase-4 (ab25898, Abcam) anti-caspase-5 (ab40887, Abcam). 2) Secondary antibody incubation: 488 goat anti-mouse IgG (A11011, Invitrogen, Carlsbad, CA, USA), 488 goat anti-rabbit IgG (A11008, Invitrogen), 555 goat anti-mouse IgG (A21422, Invitrogen) and 555 goat anti-rabbit IgG (A21428, Invitrogen) were in pairs according to the primary antibody at 37 °C for 30 min. Finally, slides were stained using Hoechst 33342 (Hoechst; ImmunoChemistry Technologies. LLC, Davis, CA, USA) for 15 min. Images were taken by a fluorescence microscope (Leica DM2000, Leica Corporation, Weztlar, Germany). The relative intensity and colocalization of two proteins (Pearson's correlation coefficient (PCC), overlap coefficient (OC) and scatterplot) were assessed by ImageJ software.

IHC for RANK (YT5881, ImmunoWay Biotechnology Company, Plano, TX, USA), RANKL (YT5404, ImmunoWay Biotechnology Company) and OPG (R1608-4, HuaBio, Hangzhou, Zhejiang, China) was performed according to the manufacture procedure (ZSGB-BIO, Beijing, China) to assess bone metabolism in dual EAP. Images were taken by a microscope (Leica DM2000). The relative positive expressions were calculated by Image J.

TUNEL staining

TUNEL staining was used to assess the spatial distribution and localization of caspase-4/-5/-11 and cell death in HAPT and EAP. The procedure was performed according to the instructions of the DeadEnd™ Fluorometric TUNEL System (Promega Co., Ltd, Madison, WI, USA). For caspase-4/-5/-11 and TUNEL dual labelling, tissues were incubated in the following sequences: permeabilization and antigen retrieval by using proteinase K for 10 min. equilibration buffer for 15 min; TdT reaction mix (rTdT enzyme and biotinylated Nucleotide Mix in equilibration buffer) for 60 min at 37 °C. After adequate washing, the specimens were incubated in 5% bovine serum albumin (BSA) at 37 °C for 60 min followed by anti-caspase-4 (ab25898, Abcam), anti-caspase-5 (ab40887, Abcam) and anti-caspase-11 (sc-374615, Santa Cruz Biotechnology) overnight at 4 °C and 594 goat anti-rabbit IgG (8889S, Cell Signalling Technology. Inc, Danvers, MA, USA) or goat anti-mouse IgG (8890S, Cell Signalling Technology) at 37 °C for 30 min. All nuclei were visualized by using Hoechst 33342 (Hoechst; ImmunoChemistry Technologies) for 15 min. Images were taken by a fluorescence microscope (Olympus Bx53, Olympus Corporation, Tokyo, Japan). The TUNEL-positive rate was calculated, and the relative intensity of caspase-4/-5/-11 and colocalization of TUNEL and caspase-4/-5/-11 (PCC, OC and scatterplot) were assessed by ImageJ [5,6].

Cell culture

THP-1 cells (ATCC® TIB-202™) were purchased from American Type Culture Collection. The cell line was identified and considered to be "identical" to the reference cell line in the Cell Bank STR database, as the STR profile yields a 100% match (GENEWIZ, Inc. Suzhou, Jiangsu, China). Cells were cultured in RPMI-1640 (Gibco, Grand Island, NY, USA) containing 10% fetal calf serum (Biowest, France), 100 U/mL penicillin and 100 μg/mL streptomycin (HyClone, UT, USA) and incubated at 37 °C in 5% CO_2. To obtain THP-1-derived macrophages, THP-1 cells were seeded at a density of 1×10^7 cells per well in a six-well culture plate and stimulated with 100 ng/mL Phorbol 12-Myristate 13-Acetate (PMA) (Sigma-Aldrich) for 24 h, followed by further incubation in RPMI medium in the absence of PMA for 24 h [7].

Lactic dehydrogenase (LDH) release assay

THP-1-derived macrophages were serum-starved for 24 h followed by treatment with 0.1~10 μg/mL *P. gingivalis* LPS (Invivogen, San Diego, CA, USA) and/or 10 μM caspase-1/-4/-5 inhibitor, Ac-FLTD-CMK (Selleck Chemicals, Houson, TX, USA) [8], at 5% CO_2 for 6 h. Cell culture supernatant was collected, and LDH release was detected using a CytoTox 96 Non-Radioactive Cytotoxicity Assay Kit (Promega) according to the manufacturer's protocol. All values represent the percentage of LDH release compared with a maximum LDH release control (Lysis Solution). The absorbance was measured using a microplate metre (SpectraMax iD3, Molecular Devices, LLC, San Jose, CA USA).

Propidium iodide (PI) staining

THP-1-derived macrophages were serum-starved for 24 h followed by treatment with 0.1~10 μg/mL *P. gingivalis* LPS and/or 10 μM caspase-1/-4/-5 inhibitor, Ac-FLTD-CMK, at 5% CO_2 for 6 h. To assess cell death, propidium iodide (PI) marks dying cells, while 496-diamidino-2-phenylindole (DAPI) stains all nuclei. Images were captured by using a fluorescence microscope (Olympus IX73, Olympus Corporation, Tokyo, Japan). The experiments were either carried out in triplicate or quadrupled in three independent experiments. The PI positive cells were calculated by Image J.

Western blot

THP-1-derived macrophages were serum-starved for 24 h followed by treatment with 1 μg/mL *P. gingivalis* LPS and/or 10 μM caspase-1/-4/-5 inhibitor Ac-FLTD-CMK at 5% CO_2 for 6 h. Cells were collected, and proteins were extracted using a Total Protein Extraction Kit (Signalway Antibody, Greenbelt, MD, USA) according to the manufacturer's instructions. Equal amounts of protein were separated using 10% SDS–PAGE (Bio–Rad) and transferred to polyvinylidene difluoride membranes (GE Healthcare Life Science, Pittsburgh, PA, USA). The membrane was incubated in blocking buffer (5% BSA in Tris-buffered saline containing 0.1% Tween 20) at room temperature for 1 h and then incubated with the following primary antibodies overnight at 4 °C: anti-caspase-1 (sc-398715, Santa Cruz Biotechnology), anti-caspase-4 (sc-56056, Santa Cruz Biotechnology), anti-caspase-5 (sc-393346, Santa Cruz Biotechnology), anti-GADMD-N (ab215203, Abcam), anti-IL-1β (ab9722, Abcam) and anti-GAPDH (Signalway Antibody). After washing, the membranes were incubated with horseradish peroxidase-conjugated secondary antibody for 1 h at 37 °C. A chemiluminescence kit (Bio-Rad) was used to visualize the immunoreactive bands. The bands were analysed by ImageJ software.

Data analysis

All data are presented as the mean ± S.D. The statistically significant difference among groups was assessed using Student's t test or one-way ANOVA with SPSS 22.0 (IBM Corp. New York, NY, USA). Comparisons between two groups were performed by Student's t test. For more than two groups, one-way ANOVA was performed. $p < 0.05$ was considered statistically significant.

References

1. Cheng, R.; Feng, Y.; Zhang, R.; Liu, W.; Lei, L.; Hu, T. The extent of pyroptosis varies in different stages of apical periodontitis. *Biochim. Biophys. Acta* **2018**, *1864*, 226–237. https://doi.org/10.1016/j.bbadis.2017.10.025.
2. Miao, N.J.; Xie, H.Y.; Xu, D.; Yin, J.Y.; Wang, Y.Z.; Wang, B.; Yin, F.; Zhou, Z.L.; Cheng, Q.; Chen, P.P.; et al. Caspase-11 promotes renal fibrosis by stimulating IL-1beta maturation via activating caspase-1. *Acta Pharmacol. Sin.* **2019**, *40*, 790–800. https://doi.org/10.1038/s41401-018-0177-5.
3. Zhang, R.; Wu, Z.; Li, M.; Yang, J.; Cheng, R.; Hu, T. Canonical and noncanonical pyroptosis are both activated in periodontal inflammation and bone resorption. *J. Periodontal. Res.* **2022**. https://doi.org/10.1111/jre.13055.
4. Xu, R.; Guo, D.; Zhou, X.; Sun, J.; Zhou, Y.; Fan, Y.; Zhou, X.; Wan, M.; Du, W.; Zheng, L. Disturbed bone remodelling activity varies in different stages of experimental, gradually progressive apical periodontitis in rats. *Int. J. Oral Sci.* **2019**, *11*, 27. https://doi.org/10.1038/s41368-019-0058-x.

5. Zeitvogel, F.; Schmid, G.; Hao, L.; Ingino, P.; Obst, M. ScatterJ: An ImageJ plugin for the evaluation of analytical microscopy datasets. *J. Microsc.* **2016**, *261*, 148–156. https://doi.org/10.1111/jmi.12187.
6. Dunn, K.W.; Kamocka, M.M.; McDonald, J.H. A practical guide to evaluating colocalization in biological microscopy. *Am. J. Physiol. Cell Physiol.* **2011**, *300*, C723–C742. https://doi.org/10.1152/ajpcell.00462.2010.
7. Zhou, P.; Li, Q.; Su, S.; Dong, W.; Zong, S.; Ma, Q.; Yang, X.; Zuo, D.; Zheng, S.; Meng, X.; et al. Interleukin 37 Suppresses M1 Macrophage Polarization Through Inhibition of the Notch1 and Nuclear Factor Kappa B Pathways. *Front. Cell Dev. Biol.* **2020**, *8*, 56. https://doi.org/10.3389/fcell.2020.00056.
8. Yang, J.; Liu, Z.; Wang, C.; Yang, R.; Rathkey, J.K.; Pinkard, O.W.; Shi, W.; Chen, Y.; Dubyak, G.R.; Abbott, D.W.; et al. Mechanism of gasdermin D recognition by inflammatory caspases and their inhibition by a gasdermin D-derived peptide inhibitor. *Proc. Natl. Acad. Sci. USA* **2018**, *115*, 6792–6797. https://doi.org/10.1073/pnas.1800562115.
