# Supplementary material for: Double-Edged Sword Effect of Pyroptosis: The Role of Caspase-1/-4/-5/-11 in Different Levels of Apical Periodontitis
Source: Biomolecules. 2022 Nov 8;12(11):1660. doi: 10.3390/biom12111660 (PMC9687662; doi:10.3390/biom12111660)
Supplement: Supplementary file 1 [file biomolecules-12-01660-s001.zip › biomolecules-1924891 - Supplementary 2 - proofed.pdf]

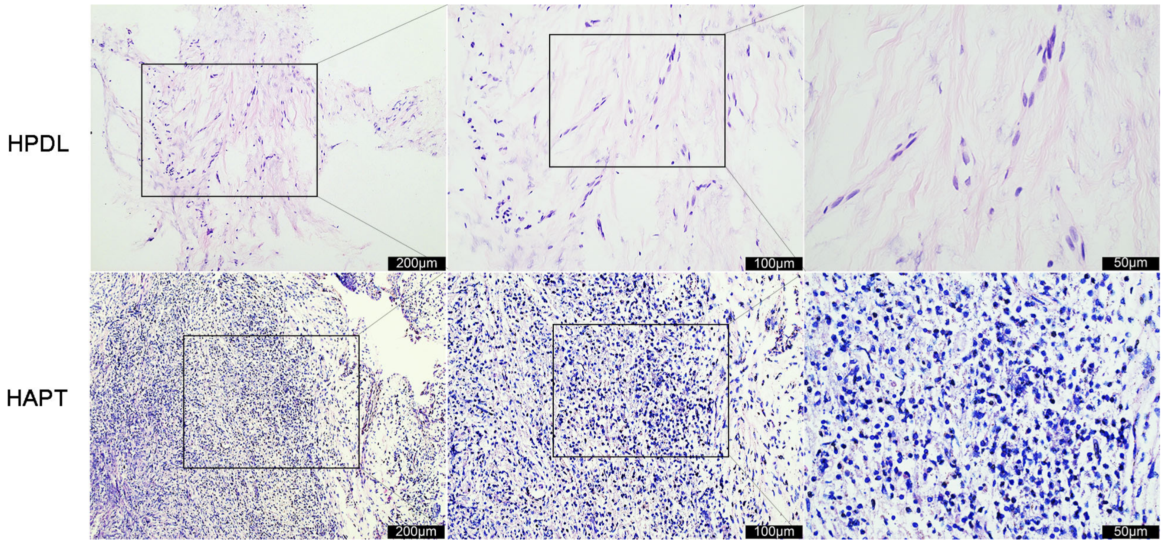

Figure S1. The HE staining of human AP. The apical lesion developed gradually (Bar, left,200 μm; middle, 100 μm; right, 50 μm).

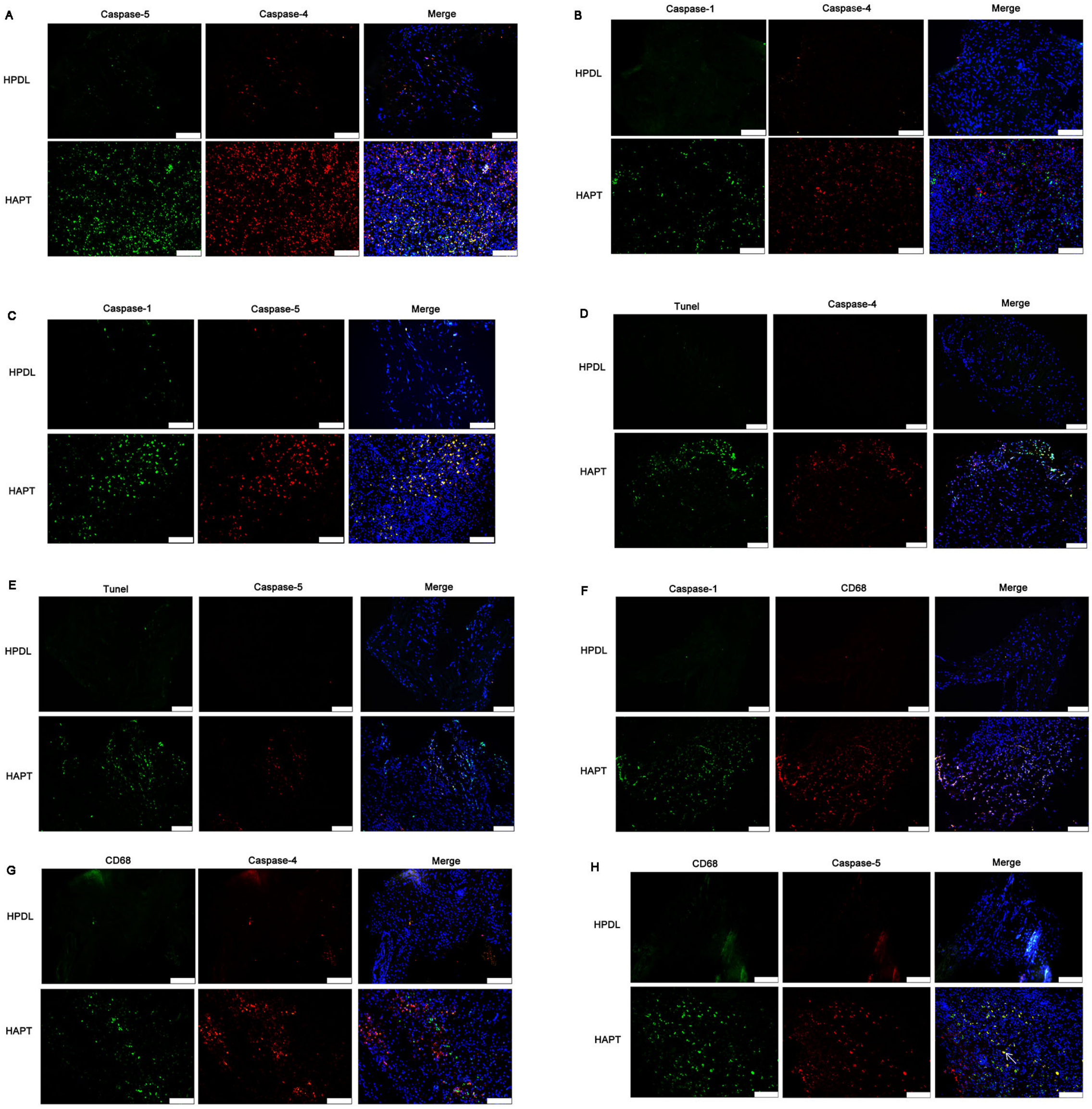

Figure S2. Different levels of pyroptosis were detected in human AP by using TUNEL staining and immunofluorescence (Bar, 100 μm). (**A**) Triple labelling of caspase-5 (green), caspase-4 (red) and Hoechst (blue). (**B**,**C**) Triple labelling of caspase-1 (green), caspase-4/-5 (red) and Hoechst (blue). (**D**,**E**) TUNEL (green), caspase-4/-5 (red) and Hoechst (blue). (**F**) Triple labelling of caspase-1 (green), CD68 (red) and Hoechst (blue). (**G**,**H**) Triple labelling of CD68 (green), caspase-4/-5 (red) and Hoechst (blue).

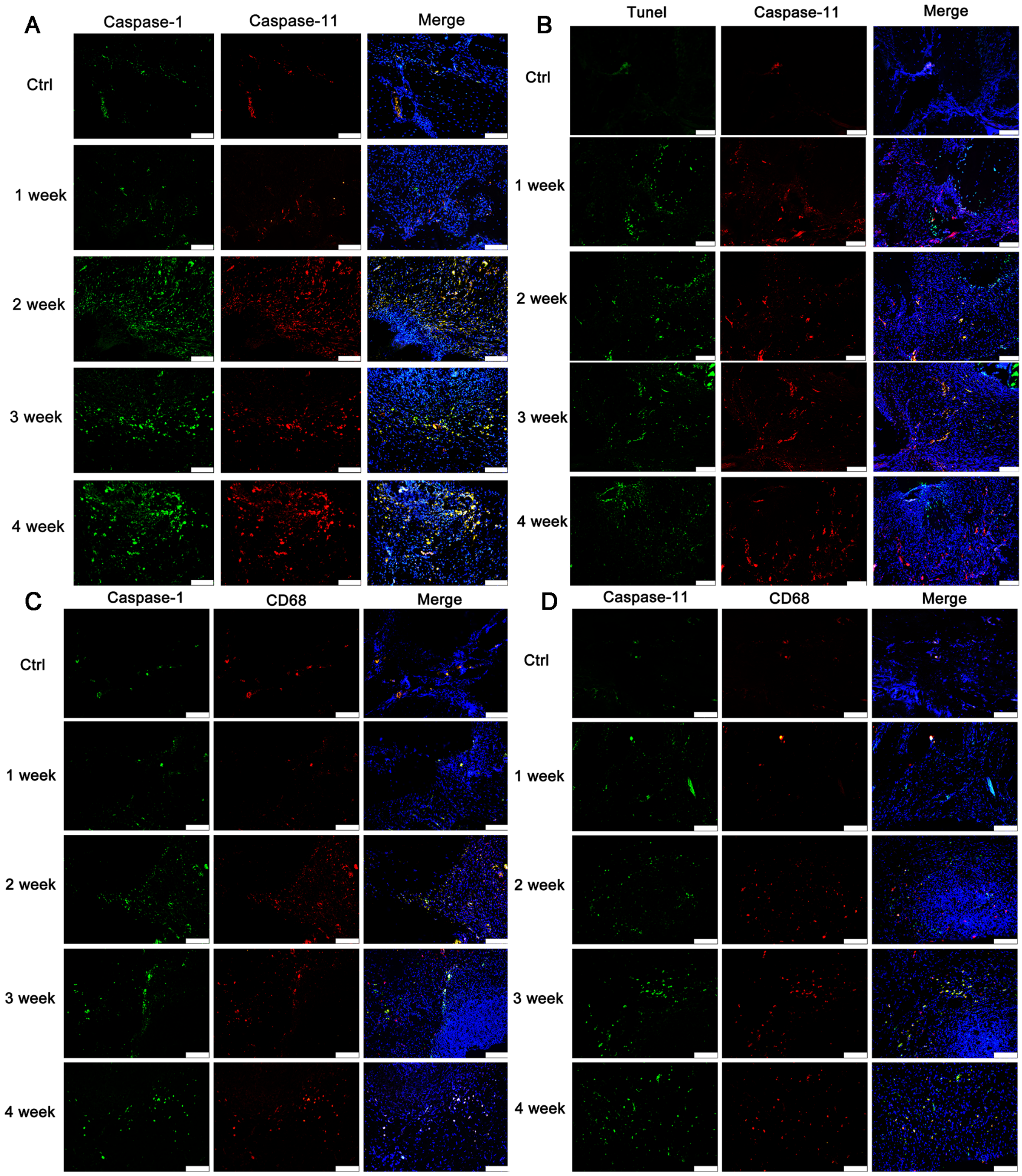

Figure S3. Pyroptosis was detected in EAP by using TUNEL staining and immunofluorescence (Bar, 100 μm). (**A**) Triple labelling of caspase-1 (green), caspase-11 (red) and Hoechst (blue). (**B**) TUNEL (green), caspase-11 (red) and Hoechst (blue) staining. (**C**,**D**) Triple labelling of caspase-1/-11 (green), CD68 (red) and Hoechst (blue).

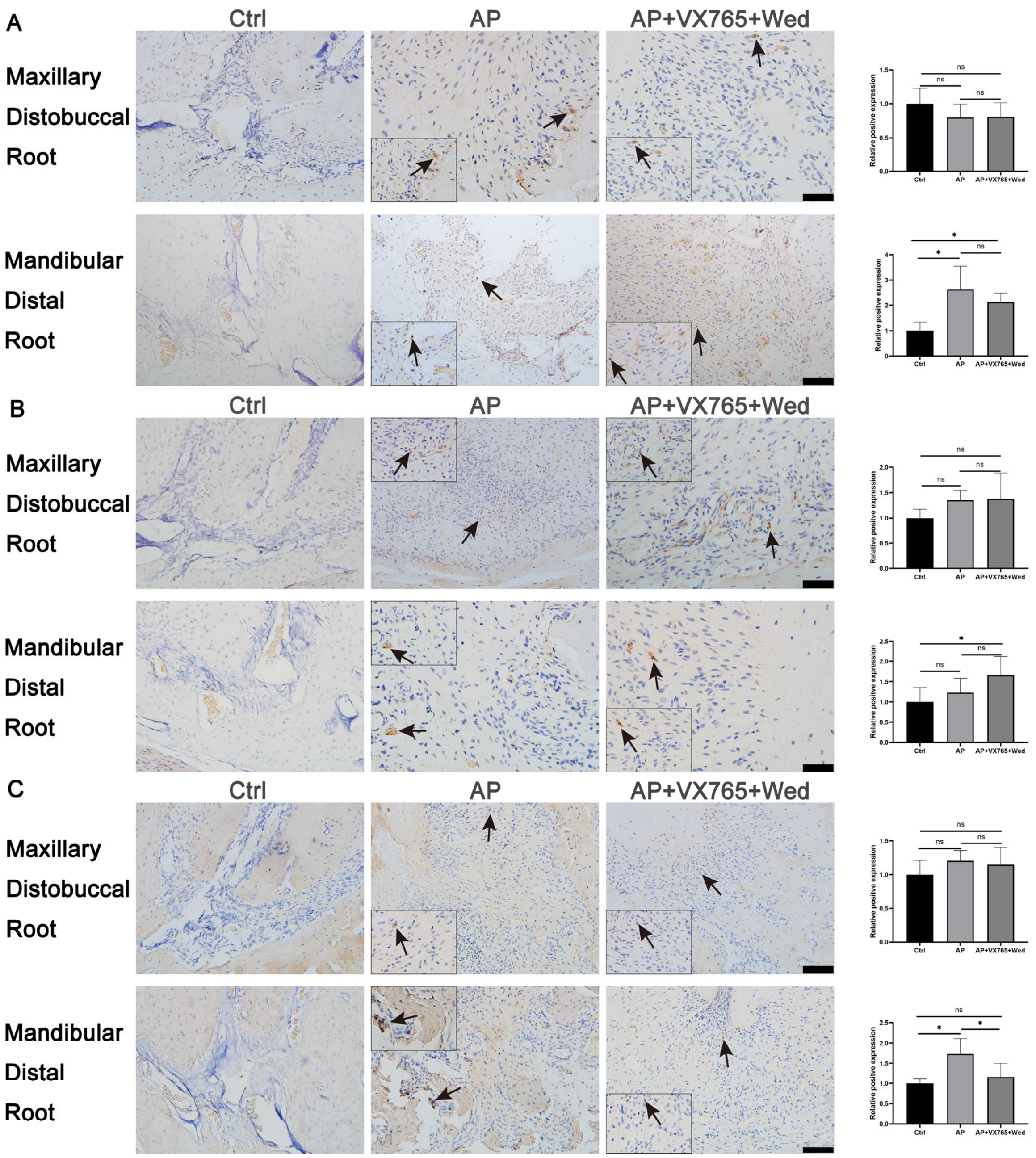

Figure S4. The expressions of RANK (A), RANKL (B) and OPG (C) were analyzed by IHC in dual EAP (Bar, 100 μm). Data are represented as the mean ± S.D. (one-way ANOVA, n = 3-5; *, p <0.05; ns, no significance).

Table S1. Proteins with significantly changed in abundance.

Protein	Protein Name	Gene Name	HAPT/HPDL	t test p value
Q8WU39	Marginal zone B- and B1-cell-specific protein	MZB1	73.600775	1.933E-05
P43490	Nicotinamide phosphoribosyltransferase	NAMPT	70.427028	0.000225
P31146	Coronin-1A	CORO1A	52.701019	0.0029972
P30740	Leukocyte elastase inhibitor	SERPINB1	41.624637	0.0007338
P05109	Protein S100-A8	S100A8	38.755916	0.0020165
Q15005	Signal peptidase complex subunit 2	SPCS2	38.560847	5.404E-05
P52209	6-phosphogluconate dehydrogenase, decarboxylating	PGD	38.241628	1.588E-05
P06702	Protein S100-A9	S100A9	35.533593	0.0068562
O60763	General vesicular transport factor p115	USO1	30.757095	0.0005094
P53999	Activated RNA polymerase II transcriptional coactivator p15	SUB1	29.086952	0.0001498
P05164	Myeloperoxidase	MPO	25.160217	0.0010001
P15311	Ezrin	EZR	25.124122	0.0401746
P02788	Lactotransferrin	LTF	22.543641	0.0002742
P04179	Superoxide dismutase [Mn], mitochondrial	SOD2	22.059352	0.0059156
P13693	Translationally-controlled tumor protein	TPT1	20.177018	3.75E-05
Q9Y4L1	Hypoxia up-regulated protein 1	HYOU1	18.014676	0.000407
P07858	Cathepsin B	CTSB	17.116424	9.165E-05
P08311	Cathepsin G	CTSG	16.506466	0.0049265
P37837	Transaldolase	TALDO1	14.905148	9.664E-06

P16152	Carbonyl reductase [NADPH] 1	CBR1	14.74873	0.0197662
P04080	Cystatin-B	CSTB	13.540216	0.0413929
Q9NYU2	UDP-glucose:glycoprotein glucosyltransferase 1	UGGT1	13.393664	4.34E-05
Q15661	Tryptase alpha/beta-1	TPSAB1	12.99679	0.0002033
P36871	Phosphoglucomutase-1	PGM1	12.705405	0.0047503
Q01518	Adenylyl cyclase-associated protein 1	CAP1	12.644455	7.294E-07
P29401	Transketolase	TKT	12.097386	0.0001156
P13796	Plastin-2	LCP1	11.59882	0.0085678
P28838	Cytosol aminopeptidase	LAP3	11.556085	0.0002241
O60664	Perilipin-3	PLIN3	11.393709	0.03006
P68366	Tubulin alpha-4A chain	TUBA4A	11.320866	0.0208807
P01861	Immunoglobulin heavy constant gamma 4	IGHG4	11.24931	7.433E-05
P49588	Alanine--tRNA ligase, cytoplasmic	AARS	11.097792	0.0284261
Q9Y6N5	Sulfide:quinone oxidoreductase, mitochondrial	SQOR	10.74536	3.727E-05
P01619	Immunoglobulin kappa variable 3-20	IGKV3-20	10.677208	0.0144154
P31949	Protein S100-A11	S100A11	10.606169	0.0497932
P08246	Neutrophil elastase	ELANE	10.253835	0.0042579
Q9Y6A9	Signal peptidase complex subunit 1	SPCS1	9.83963	0.0295808
P01860	Immunoglobulin heavy constant gamma 3	IGHG3	9.435937	0.0161965
P23526	Adenosylhomocysteinase	AHCY	9.3346937	3.283E-05
P06744	Glucose-6-phosphate isomerase	GPI	9.2842367	8.82E-05
Q99613	Eukaryotic translation initiation factor 3 subunit C	EIF3C	9.2211055	0.0198939

P23381	Tryptophan--tRNA ligase, cytoplasmic	WARS	8.5649299	0.0008929
Q06323	Proteasome activator complex subunit 1	PSME1	8.4992001	0.0070225
P01859	Immunoglobulin heavy constant gamma 2	IGHG2	8.2418995	0.0061624
P15153	Ras-related C3 botulinum toxin substrate 2	RAC2	8.2268187	0.0004186
P01780	Immunoglobulin heavy variable 3-7	IGHV3-7	8.2079999	0.0135741
P13667	Protein disulfide-isomerase A4	PDIA4	7.9431141	1.095E-06
Q14697	Neutral alpha-glucosidase AB	GANAB	7.8897558	0.0005848
P02792	Ferritin light chain	FTL	7.6900265	0.0473671
P0DOY3	Immunoglobulin lambda constant 3	IGLC3	7.3452092	0.0042692
Q13162	Peroxiredoxin-4	PRDX4	7.1493426	0.0003845
O00410	Importin-5	IPO5	7.1455565	0.0003615
P60228	Eukaryotic translation initiation factor 3 subunit E	EIF3E	7.132173	0.0009216
P38606	V-type proton ATPase catalytic subunit A	ATP6V1A	7.0945009	0.0002255
P63241	Eukaryotic translation initiation factor 5A-1	EIF5A	6.6097069	0.0226423
P06737	Glycogen phosphorylase, liver form	PYGL	6.5626842	0.0204444
P40121	Macrophage-capping protein	CAPG	6.554361	0.0004305
Q86UX7	Fermitin family homolog 3	FERMT3	6.5370564	0.0003695
Q9Y673	Dolichyl-phosphate beta-glucosyltransferase	ALG5	6.5344135	0.0003731
P49257	Protein ERGIC-53	LMAN1	6.4693578	0.0006152
P46783	40S ribosomal protein S10	RPS10	6.3688194	0.0002623
Q9NZL9	Methionine adenosyltransferase 2 subunit beta	MAT2B	6.3070008	0.007851

P0DOX8	Immunoglobulin lambda-1 light chain		6.2995787	0.00108 63
P0DOX2	Immunoglobulin alpha-2 heavy chain		6.2426829	4.052E-06
P07814	Bifunctional glutamate/proline--tRNA ligase	EPRS	6.2190244	0.00036 24
P00338	L-lactate dehydrogenase A chain	LDHA	6.1202714	0.00012 28
Q969H8	Myeloid-derived growth factor	MYDGF	6.0473302	0.00041 6
O00231	26S proteasome non-ATPase regulatory subunit 11	PSMD11	5.990236	6.358E-05
P14625	Endoplasmin	HSP90B1	5.9273423	2.33E-05
P18669	Phosphoglycerate mutase 1	PGAM1	5.760439	0.00018 42
P11940	Polyadenylate-binding protein 1	PABPC1	5.7394847	0.00120 19
P02794	Ferritin heavy chain	FTH1	5.639196	0.03441 75
P60842	Eukaryotic initiation factor 4A-I	EIF4A1	5.5914235	0.00109 75
P28062	Proteasome subunit beta type-8	PSMB8	5.5759238	6.471E-05
P09211	Glutathione S-transferase P	GSTP1	5.4149992	0.00195 88
P0DOX5	Immunoglobulin gamma-1 heavy chain		5.3668208	0.00024 99
P14868	Aspartate--tRNA ligase, cytoplasmic	DARS	5.3453254	9.208E-05
P59666	Neutrophil defensin 3	DEFA3	5.2954652	0.00028 7
Q9Y262	Eukaryotic translation initiation factor 3 subunit L	EIF3L	5.2876906	0.01694 32
P14550	Aldo-keto reductase family 1 member A1	AKR1A1	5.2820822	0.00014 27
O94979	Protein transport protein Sec31A	SEC31A	5.2411244	0.00013 14
A2NJV5	Immunoglobulin kappa variable 2-29	IGKV2-29	5.221547	0.00096 27
Q8NBS9	Thioredoxin domain-containing protein 5	TXNDC5	5.0673202	1.556E-11

P69905	Hemoglobin subunit alpha	HBA1	4.9505249	0.0495936
Q12907	Vesicular integral-membrane protein VIP36	LMAN2	4.8760096	1.065E-06
P06733	Alpha-enolase	ENO1	4.8703206	0.0004413
P34932	Heat shock 70 kDa protein 4	HSPA4	4.834249	0.0083224
P61088	Ubiquitin-conjugating enzyme E2 N	UBE2N	4.8328023	0.0007582
P13639	Elongation factor 2	EEF2	4.7161194	0.0005376
P26641	Elongation factor 1-gamma	EEF1G	4.6590456	0.0005101
Q13200	26S proteasome non-ATPase regulatory subunit 2	PSMD2	4.6273915	0.0197949
P68871	Hemoglobin subunit beta	HBB	4.6155509	0.0100538
P42224	Signal transducer and activator of transcription 1-alpha/beta	STAT1	4.5724023	0.0004091
P78417	Glutathione S-transferase omega-1	GSTO1	4.5110559	0.0004952
Q92499	ATP-dependent RNA helicase DDX1	DDX1	4.503051	0.0006076
P0DOX7	Immunoglobulin kappa light chain		4.4971205	0.0002205
P52565	Rho GDP-dissociation inhibitor 1	ARHGDIA	4.4851636	0.0002937
Q9H299	SH3 domain-binding glutamic acid-rich-like protein 3	SH3BGRL3	4.3783989	0.0038815
P42766	60S ribosomal protein L35	RPL35	4.3709642	0.0013556
P02787	Serotransferrin	TF	4.3665127	0.0006204
P61626	Lysozyme C	LYZ	4.3610336	0.000172
P24539	ATP synthase F(0) complex subunit B1, mitochondrial	ATP5PB	4.3514192	0.0009089
Q14152	Eukaryotic translation initiation factor 3 subunit A	EIF3A	4.345158	0.0032043
Q9BY50	Signal peptidase complex catalytic subunit SEC11C	SEC11C	4.3350581	0.000826

Q8WUM4	Programmed cell death 6-interacting protein	PDCD6IP	4.3309184	0.0104483
Q06830	Peroxiredoxin-1	PRDX1	4.3127263	0.0005874
Q96G03	Phosphoglucomutase-2	PGM2	4.2873373	0.0370353
Q96I99	Succinate--CoA ligase [GDP-forming] subunit beta, mitochondrial	SUCLG2	4.2519636	0.0024041
Q9UQ80	Proliferation-associated protein 2G4	PA2G4	4.2114442	0.0026308
Q13423	NAD(P) transhydrogenase, mitochondrial	NNT	4.1897052	0.000272
P80303	Nucleobindin-2	NUCB2	4.0750013	0.0028242
Q15629	Translocating chain-associated membrane protein 1	TRAM1	4.074192	6.163E-06
Q01105	Protein SET	SET	4.068218	0.0014275
Q15363	Transmembrane emp24 domain-containing protein 2	TMED2	4.0619334	0.0155689
P61009	Signal peptidase complex subunit 3	SPCS3	4.0313698	5.534E-05
Q99497	Protein/nucleic acid deglycase DJ-1	PARK7	4.0132539	9.115E-05
Q8TCJ2	Dolichyl-diphosphooligosaccharide--protein glycosyltransferase subunit STT3B	STT3B	4.0050487	0.0002942
P16284	Platelet endothelial cell adhesion molecule	PECAM1	3.93506	0.0004276
P07737	Profilin-1	PFN1	3.9059091	1.742E-05
P60900	Proteasome subunit alpha type-6	PSMA6	3.876383	6.396E-05
P11021	Endoplasmic reticulum chaperone BiP	HSPA5	3.8703793	9.386E-08
P40925	Malate dehydrogenase, cytoplasmic	MDH1	3.8623106	0.0004153
P10606	Cytochrome c oxidase subunit 5B, mitochondrial	COX5B	3.8527581	5.211E-05
Q9Y4P3	Transducin beta-like protein 2	TBL2	3.8283591	0.0035964
P18031	Tyrosine-protein phosphatase non-receptor type 1	PTPN1	3.8193405	0.0114552

P02042	Hemoglobin subunit delta	HBD	3.817894	0.0424393
P00918	Carbonic anhydrase 2	CA2	3.7592212	0.0075677
Q00688	Peptidyl-prolyl cis-trans isomerase FKBP3	FKBP3	3.7366787	0.016872
Q9NYL4	Peptidyl-prolyl cis-trans isomerase FKBP11	FKBP11	3.7008754	8.998E-06
Q16658	Fascin	FSCN1	3.6768083	0.0032752
P38117	Electron transfer flavoprotein subunit beta	ETFB	3.601672	0.0030066
Q5JPE7	Nodal modulator 2	NOMO2	3.5454713	0.0381503
P18621	60S ribosomal protein L17	RPL17	3.5144572	0.0018083
P07203	Glutathione peroxidase 1	GPX1	3.4680112	0.003941
P62847	40S ribosomal protein S24	RPS24	3.4562031	0.0039255
Q6UW68	Transmembrane protein 205	TMEM205	3.4360973	0.000187
Q15181	Inorganic pyrophosphatase	PPA1	3.4356695	0.0002708
P27797	Calreticulin	CALR	3.4020828	5.954E-05
P43307	Translocon-associated protein subunit alpha	SSR1	3.3939477	0.0012934
P46778	60S ribosomal protein L21	RPL21	3.3625001	0.0361917
P48735	Isocitrate dehydrogenase [NADP], mitochondrial	IDH2	3.3617681	0.0002563
P01876	Immunoglobulin heavy constant alpha 1	IGHA1	3.3604866	1.314E-05
P00450	Ceruloplasmin	CP	3.3280687	0.0389644
P30044	Peroxiredoxin-5, mitochondrial	PRDX5	3.3234354	0.0001519
P35606	Coatomer subunit beta	COPB2	3.3192585	0.0006811
P17980	26S proteasome regulatory subunit 6A	PSMC3	3.3187044	0.0007494

Q14764	Major vault protein	MVP	3.3040357	0.00208 72
P07384	Calpain-1 catalytic subunit	CAPN1	3.2975057	0.00609 66
P09622	Dihydrolipoyl dehydrogenase, mitochondrial	DLD	3.2694649	0.00187 08
P28066	Proteasome subunit alpha type-5	PSMA5	3.2633422	0.00083 52
P25788	Proteasome subunit alpha type-3	PSMA3	3.2506959	0.00178 74
P49591	Serine--tRNA ligase, cytoplasmic	SARS	3.2502006	9.771E-05
P62937	Peptidyl-prolyl cis-trans isomerase A	PPIA	3.2400909	9.374E-05
P51571	Translocon-associated protein subunit delta	SSR4	3.1823168	0.00248 64
P62266	40S ribosomal protein S23	RPS23	3.1816691	0.00455 21
O00299	Chloride intracellular channel protein 1	CLIC1	3.1470996	0.00013 3
P30043	Flavin reductase (NADPH)	BLVRB	3.0969382	0.00168 95
P50395	Rab GDP dissociation inhibitor beta	GDI2	3.0950451	0.00062 51
P07237	Protein disulfide-isomerase	P4HB	3.0900945	3.371E-08
P54886	Delta-1-pyrroline-5-carboxylate synthase	ALDH18A 1	3.081093	0.00131 34
P05386	60S acidic ribosomal protein P1	RPLP1	3.0706624	0.00116 11
P01903	HLA class II histocompatibility antigen, DR alpha chain	HLA-DRA	3.0589762	0.00464 04
P32119	Peroxiredoxin-2	PRDX2	3.0531162	0.01021 43
P22392	Nucleoside diphosphate kinase B	NME2	3.0434152	2.563E-05
P27695	DNA-(apurinic or apyrimidinic site) lyase	APEX1	3.0413317	0.00462 42
P06703	Protein S100-A6	S100A6	3.0227937	0.00013 27
P04114	Apolipoprotein B-100	APOB	3.0089853	0.04475 84

P55884	Eukaryotic translation initiation factor 3 subunit B	EIF3B	3.0033626	0.0009137
Q8TCT9	Minor histocompatibility antigen H13	HM13	2.994904	3.005E-05
O75083	WD repeat-containing protein 1	WDR1	2.991808	0.000745
P14618	Pyruvate kinase PKM	PKM	2.9849135	0.0095748
O15143	Actin-related protein 2/3 complex subunit 1B	ARPC1B	2.959526	0.0025649
Q16795	NADH dehydrogenase [ubiquinone] 1 alpha subcomplex subunit 9, mitochondrial	NDUFA9	2.9342472	0.0487469
Q15084	Protein disulfide-isomerase A6	PDIA6	2.9213685	5.851E-06
Q13838	Spliceosome RNA helicase DDX39B	DDX39B	2.9205031	0.0458455
Q9H8H3	Methyltransferase-like protein 7A	METTL7A	2.9114085	3.151E-05
P21281	V-type proton ATPase subunit B, brain isoform	ATP6V1B2	2.9086663	0.0360833
P60174	Triosephosphate isomerase	TPI1	2.9010358	5.16E-05
P61956	Small ubiquitin-related modifier 2	SUMO2	2.8900095	0.0002149
P48444	Coatomer subunit delta	ARCN1	2.8853692	0.0021342
O75874	Isocitrate dehydrogenase [NADP] cytoplasmic	IDH1	2.8715063	0.0005001
O75390	Citrate synthase, mitochondrial	CS	2.8462291	0.0007785
P23528	Cofilin-1	CFL1	2.8327009	5.887E-05
P00915	Carbonic anhydrase 1	CA1	2.7914556	0.0384786
O14818	Proteasome subunit alpha type-7	PSMA7	2.7878002	0.0002635
P37802	Transgelin-2	TAGLN2	2.7853037	0.0059497
Q15907	Ras-related protein Rab-11B	RAB11B	2.7771599	0.0001824
P07339	Cathepsin D	CTSD	2.7627314	2.482E-05

P26038	Moesin	MSN	2.7607159	6.791E-06
P07900	Heat shock protein HSP 90-alpha	HSP90AA1	2.7447459	6.852E-06
P62269	40S ribosomal protein S18	RPS18	2.7410692	6.974E-05
P54819	Adenylate kinase 2, mitochondrial	AK2	2.7386689	0.0105994
P62826	GTP-binding nuclear protein Ran	RAN	2.730553	0.0010166
O00571	ATP-dependent RNA helicase DDX3X	DDX3X	2.7205147	0.0232375
O75955	Flotillin-1	FLOT1	2.7185974	0.0337112
P01834	Immunoglobulin kappa constant	IGKC	2.7180315	0.0008389
P04839	Cytochrome b-245 heavy chain	CYBB	2.6790785	0.027264
P08238	Heat shock protein HSP 90-beta	HSP90AB1	2.6738609	0.0008902
Q15365	Poly(rC)-binding protein 1	PCBP1	2.6722543	0.0084138
P46977	Dolichyl-diphosphooligosaccharide--protein glycosyltransferase subunit STT3A	STT3A	2.6363681	0.00025
P00505	Aspartate aminotransferase, mitochondrial	GOT2	2.6305324	0.0038744
P62081	40S ribosomal protein S7	RPS7	2.6196227	0.0140822
P68104	Elongation factor 1-alpha 1	EEF1A1	2.5972493	0.0003343
P46782	40S ribosomal protein S5	RPS5	2.5904512	0.0030952
P00738	Haptoglobin	HP	2.5693137	0.0219679
Q99798	Aconitate hydratase, mitochondrial	ACO2	2.561191	5.699E-05
P55036	26S proteasome non-ATPase regulatory subunit 4	PSMD4	2.5444113	0.0166582
Q8IYT4	Katanin p60 ATPase-containing subunit A-like 2	KATNAL2	2.53406	0.0002774
P46776	60S ribosomal protein L27a	RPL27A	2.531706	0.0004131

Q14240	Eukaryotic initiation factor 4A-II	EIF4A2	2.5183027	0.007942
P62841	40S ribosomal protein S15	RPS15	2.4969131	0.0061674
P17066	Heat shock 70 kDa protein 6	HSPA6	2.4966005	0.0007125
P19367	Hexokinase-1	HK1	2.4962878	0.0067881
P14314	Glucosidase 2 subunit beta	PRKCSH	2.4729969	0.0001029
P29692	Elongation factor 1-delta	EEF1D	2.4690844	7.124E-05
P60866	40S ribosomal protein S20	RPS20	2.4677413	0.000828
Q07955	Serine/arginine-rich splicing factor 1	SRSF1	2.461538	0.0057149
O00303	Eukaryotic translation initiation factor 3 subunit F	EIF3F	2.4474672	0.0095888
P84077	ADP-ribosylation factor 1	ARF1	2.4233542	0.0002451
P62277	40S ribosomal protein S13	RPS13	2.414822	0.0058763
P12236	ADP/ATP translocase 3	SLC25A6	2.4109214	0.0005217
P24534	Elongation factor 1-beta	EEF1B2	2.4097748	2.586E-05
Q02543	60S ribosomal protein L18a	RPL18A	2.4078098	0.0021334
Q9Y3Z3	Deoxynucleoside triphosphate triphosphohydrolase SAMHD1	SAMHD1	2.4055654	0.0058567
P26639	Threonine--tRNA ligase 1, cytoplasmic	TARS1	2.4046771	0.0093885
Q00765	Receptor expression-enhancing protein 5	REEP5	2.3966654	0.0002488
O15144	Actin-related protein 2/3 complex subunit 2	ARPC2	2.390496	2.219E-06
Q96DZ1	Endoplasmic reticulum lectin 1	ERLEC1	2.3896669	0.0032245
P17858	ATP-dependent 6-phosphofructokinase, liver type	PFKL	2.3809613	0.0045388
P56192	Methionine--tRNA ligase, cytoplasmic	MARS	2.3613978	0.0094101

P22314	Ubiquitin-like modifier-activating enzyme 1	UBA1	2.354782	0.00298 02
P62899	60S ribosomal protein L31	RPL31	2.3482596	0.00619 65
O15258	Protein RER1	RER1	2.3332746	0.00033 46
P62314	Small nuclear ribonucleoprotein Sm D1	SNRPD1	2.3312236	0.00699 23
P02766	Transthyretin	TTR	2.3157039	0.00581 28
P42126	Enoyl-CoA delta isomerase 1, mitochondrial	ECI1	2.3105009	0.01993 6
P05387	60S acidic ribosomal protein P2	RPLP2	2.2975756	0.00212 67
P62140	Serine/threonine-protein phosphatase PP1-beta catalytic subunit	PPP1CB	2.2886307	0.01946 07
P07195	L-lactate dehydrogenase B chain	LDHB	2.2880919	7.46E-06
P61313	60S ribosomal protein L15	RPL15	2.2802529	0.00113 7
P12429	Annexin A3	ANXA3	2.2760979	0.01108 41
P53396	ATP-citrate synthase	ACLY	2.2753431	0.00106 38
P00390	Glutathione reductase, mitochondrial	GSR	2.2575626	0.00016 79
P50914	60S ribosomal protein L14	RPL14	2.2552538	0.00403 25
Q9BVK6	Transmembrane emp24 domain-containing protein 9	TMED9	2.254276	7.248E-05
P08559	Pyruvate dehydrogenase E1 component subunit alpha, somatic form, mitochondrial	PDHA1	2.2447648	0.01599 53
P11766	Alcohol dehydrogenase class-3	ADH5	2.2424478	0.00162 93
P18085	ADP-ribosylation factor 4	ARF4	2.2420223	0.00257 63
O95571	Persulfide dioxygenase ETHE1, mitochondrial	ETHE1	2.234562	0.01637 53
P63279	SUMO-conjugating enzyme UBC9	UBE2I	2.2341173	1.096E-05
P53621	Coatomer subunit alpha	COPA	2.2295282	0.01503 77

P59998	Actin-related protein 2/3 complex subunit 4	ARPC4	2.2216962	0.0002913
P61353	60S ribosomal protein L27	RPL27	2.2173375	0.0076722
P42785	Lysosomal Pro-X carboxypeptidase	PRCP	2.2117504	0.0269004
P18077	60S ribosomal protein L35a	RPL35A	2.2112628	0.0030974
P63104	14-3-3 protein zeta/delta	YWHAZ	2.2109303	0.0109284
P39023	60S ribosomal protein L3	RPL3	2.1765439	0.0032146
P61604	10 kDa heat shock protein, mitochondrial	HSPE1	2.1706481	5.594E-06
P55072	Transitional endoplasmic reticulum ATPase	VCP	2.1699691	0.0001738
P08708	40S ribosomal protein S17	RPS17	2.1669901	0.004533
Q96TA2	ATP-dependent zinc metalloprotease YME1L1	YME1L1	2.1645598	0.0127668
P63244	Receptor of activated protein C kinase 1	RACK1	2.1594543	0.0007563
P52907	F-actin-capping protein subunit alpha-1	CAPZA1	2.1574074	0.0008706
P04844	Dolichyl-diphosphooligosaccharide--protein glycosyltransferase subunit 2	RPN2	2.1546472	0.0003806
P62750	60S ribosomal protein L23a	RPL23A	2.1534017	0.0194237
P26373	60S ribosomal protein L13	RPL13	2.1434457	0.0100313
P07954	Fumarate hydratase, mitochondrial	FH	2.1398272	0.0223444
P30101	Protein disulfide-isomerase A3	PDIA3	2.1391356	7.668E-05
P05141	ADP/ATP translocase 2	SLC25A5	2.1355855	0.0111569
P04406	Glyceraldehyde-3-phosphate dehydrogenase	GAPDH	2.1297713	0.0001304
P49755	Transmembrane emp24 domain-containing protein 10	TMED10	2.1287055	0.0019657
P62263	40S ribosomal protein S14	RPS14	2.1214117	0.0096586

P46459	Vesicle-fusing ATPase	NSF	2.1193565	0.0063359
P04075	Fructose-bisphosphate aldolase A	ALDOA	2.1171588	0.0018304
P62917	60S ribosomal protein L8	RPL8	2.1157471	0.007905
O94874	E3 UFM1-protein ligase 1	UFL1	2.1032303	0.0147787
P61158	Actin-related protein 3	ACTR3	2.1021146	9.875E-05
P47985	Cytochrome b-c1 complex subunit Rieske, mitochondrial	UQCRFS1	2.0994638	0.0031365
P84098	60S ribosomal protein L19	RPL19	2.0970711	0.0111879
P08754	Guanine nucleotide-binding protein G(i) subunit alpha	GNAI3	2.0964713	0.0023648
Q9Y678	Coatomer subunit gamma-1	COPG1	2.0941947	4.424E-05
P61586	Transforming protein RhoA	RHOA	2.0911153	0.0249148
P09874	Poly [ADP-ribose] polymerase 1	PARP1	2.0894553	9.928E-05
Q01844	RNA-binding protein EWS	EWSR1	2.07409	0.0013501
P46781	40S ribosomal protein S9	RPS9	2.0652812	0.002394
P30086	Phosphatidylethanolamine-binding protein 1	PEBP1	2.0635239	0.0006242
P02790	Hemopexin	HPX	2.0624552	0.0144619
P39019	40S ribosomal protein S19	RPS19	2.0509628	0.0105098
Q9Y2Q3	Glutathione S-transferase kappa 1	GSTK1	2.0444665	7.476E-05
P30040	Endoplasmic reticulum resident protein 29	ERP29	2.0426594	7.817E-06
Q02218	2-oxoglutarate dehydrogenase, mitochondrial	OGDH	2.0399329	0.0208878
P46777	60S ribosomal protein L5	RPL5	2.0309691	0.0005855
P00558	Phosphoglycerate kinase 1	PGK1	2.0220844	0.0001737

P62701	40S ribosomal protein S4, X isoform	RPS4X	2.0159835	0.0008074
P01889	HLA class I histocompatibility antigen, B alpha chain	HLA-B	2.0159345	0.0009168
P23284	Peptidyl-prolyl cis-trans isomerase B	PPIB	2.0125696	8.741E-05
O95831	Apoptosis-inducing factor 1, mitochondrial	AIFM1	2.0052223	0.0206853
Q7Z406	Myosin-14	MYH14	0.4991192	0.0001155
Q9UHB6	LIM domain and actin-binding protein 1	LIMA1	0.4979601	0.0030418
P15144	Aminopeptidase N	ANPEP	0.4970562	0.0017357
O75475	PC4 and SFRS1-interacting protein	PSIP1	0.4964193	0.0069835
P53680	AP-2 complex subunit sigma	AP2S1	0.4928498	0.0349334
Q9Y385	Ubiquitin-conjugating enzyme E2 J1	UBE2J1	0.4912774	0.0086809
Q969G5	Caveolae-associated protein 3	CAVIN3	0.4886516	0.0023916
Q13951	Core-binding factor subunit beta	CBFB	0.48576	0.0010971
Q9UBG0	C-type mannose receptor 2	MRC2	0.4803728	0.0067401
O15460	Prolyl 4-hydroxylase subunit alpha-2	P4HA2	0.4780575	0.0237678
O94832	Unconventional myosin-Id	MYO1D	0.4776183	0.0030397
Q13642	Four and a half LIM domains protein 1	FHL1	0.4765289	0.0288705
P18564	Integrin beta-6	ITGB6	0.4757425	0.0003786
Q99584	Protein S100-A13	S100A13	0.4745598	0.0069388
Q9Y3E1	Hepatoma-derived growth factor-related protein 3	HDGFL3	0.4744146	0.0015771
P07942	Laminin subunit beta-1	LAMB1	0.4741816	0.0001061
P23634	Plasma membrane calcium-transporting ATPase 4	ATP2B4	0.4740914	0.0320048

Q02252	Methylmalonate-semialdehyde dehydrogenase [acylating], mitochondrial	ALDH6A1	0.4717019	0.00010 24
Q92614	Unconventional myosin-XVIIIa	MYO18A	0.4714693	0.00023
P00488	Coagulation factor XIII A chain	F13A1	0.4674698	1.739E-06
Q9NUV 7	Serine palmitoyltransferase 3	SPTLC3	0.4673972	0.01217 86
P21589	5-nucleotidase	NT5E	0.4669405	0.00327 25
P07099	Epoxide hydrolase 1	EPHX1	0.4650573	0.00041 46
Q02094	Ammonium transporter Rh type A	RHAG	0.4542955	0.00181 88
P04004	Vitronectin	VTN	0.4528513	8.121E-06
P00734	Prothrombin	F2	0.4510706	0.01658 37
P02775	Platelet basic protein	PPBP	0.4472895	0.02322 47
Q6ZMZ 3	Nesprin-3	SYNE3	0.4441017	0.02399 91
P02730	Band 3 anion transport protein	SLC4A1	0.4432032	0.00503 82
Q99715	Collagen alpha-1(XII) chain	COL12A1	0.442164	0.00499 41
Q06828	Fibromodulin	FMOD	0.4407739	0.00752 49
P50454	Serpin H1	SERPINH 1	0.4385601	0.00235 52
P11047	Laminin subunit gamma-1	LAMC1	0.4384807	3.707E-08
P02545	Prelamin-A/C	LMNA	0.4358336	0.00033 43
Q13885	Tubulin beta-2A chain	TUBB2A	0.4337762	0.00034 73
Q96D15	Reticulocalbin-3	RCN3	0.4305618	0.00323 47
P55290	Cadherin-13	CDH13	0.4287927	0.01628 79
P11277	Spectrin beta chain, erythrocytic	SPTB	0.4284297	0.00568 43
Q14112	Nidogen-2	NID2	0.4205837	0.00149 33

Q6PCB0	von Willebrand factor A domain-containing protein 1	VWA1	0.4205364	0.0188975
Q9BZQ8	Protein Niban 1	NIBAN1	0.4169498	0.015985
P19827	Inter-alpha-trypsin inhibitor heavy chain H1	ITIH1	0.4149151	0.0002578
O94973	AP-2 complex subunit alpha-2	AP2A2	0.4128513	0.0098284
P21810	Biglycan	BGN	0.4102267	0.0036786
P12109	Collagen alpha-1(VI) chain	COL6A1	0.4058055	0.0011442
Q96S97	Myeloid-associated differentiation marker	MYADM	0.4051906	0.0001441
Q9HBJ7	Ubiquitin carboxyl-terminal hydrolase 29	USP29	0.4042571	0.0007164
Q5SSJ5	Heterochromatin protein 1-binding protein 3	HP1BP3	0.4037154	7.63E-06
Q9BWM7	Sideroflexin-3	SFXN3	0.4034959	3.173E-06
O43427	Acidic fibroblast growth factor intracellular-binding protein	FIBP	0.3954377	0.0212381
P29972	Aquaporin-1	AQP1	0.3952924	0.0001288
Q02952	A-kinase anchor protein 12	AKAP12	0.3946312	0.0153838
Q15582	Transforming growth factor-beta-induced protein ig-h3	TGFBI	0.3870561	1.542E-06
P10909	Clusterin	CLU	0.3857868	0.0001972
Q02809	Procollagen-lysine,2-oxoglutarate 5-dioxygenase 1	PLOD1	0.3830589	0.0302922
Q8TF66	Leucine-rich repeat-containing protein 15	LRRC15	0.3712947	0.0208837
Q16777	Histone H2A type 2-C	HIST2H2AC	0.3702771	0.0173377
Q9NVA2	Septin-11	SEPTIN11	0.3697175	1.826E-05
Q9BTV4	Transmembrane protein 43	TMEM43	0.3678437	0.0010264
Q4V9L6	Transmembrane protein 119	TMEM119	0.3650857	0.0040406

O75531	Barrier-to-autointegration factor	BANF1	0.3620339	0.0004207
P36955	Pigment epithelium-derived factor	SERPINF1	0.3549291	0.0008135
P19823	Inter-alpha-trypsin inhibitor heavy chain H2	ITIH2	0.3533897	8.788E-05
P02679	Fibrinogen gamma chain	FGG	0.3524694	0.0113525
P16157	Ankyrin-1	ANK1	0.348183	0.0017567
P09382	Galectin-1	LGALS1	0.344762	4.1E-05
O43491	Band 4.1-like protein 2	EPB41L2	0.3377186	0.0002974
O94905	Erlin-2	ERLIN2	0.3374587	2.215E-05
Q07954	Prolow-density lipoprotein receptor-related protein 1	LRP1	0.3244782	0.0003284
P46939	Utrophin	UTRN	0.320726	3.692E-05
P16452	Erythrocyte membrane protein band 4.2	EPB42	0.3187772	0.0338849
P02549	Spectrin alpha chain, erythrocytic 1	SPTA1	0.3134917	0.0049848
Q9P2B2	Prostaglandin F2 receptor negative regulator	PTGFRN	0.311552	0.0166593
P36269	Glutathione hydrolase 5 proenzyme	GGT5	0.3109684	0.0001301
P78347	General transcription factor II-I	GTF2I	0.3085747	0.0027381
Q12797	Aspartyl/asparaginyl beta-hydroxylase	ASPH	0.307644	0.0173955
P02675	Fibrinogen beta chain	FGB	0.3047035	0.0079763
P07585	Decorin	DCN	0.3018404	2.183E-05
P12107	Collagen alpha-1(XI) chain	COL11A1	0.2962688	0.0002464
Q9Y5X1	Sorting nexin-9	SNX9	0.2958304	0.0001664
P60033	CD81 antigen	CD81	0.2925763	0.0178184
Q9NZM1	Myoferlin	MYOF	0.2921274	0.0001436

P02671	Fibrinogen alpha chain	FGA	0.2920056	0.0062079
P12110	Collagen alpha-2(VI) chain	COL6A2	0.2890292	2.917E-07
Q8IUX7	Adipocyte enhancer-binding protein 1	AEBP1	0.2804794	0.0002751
P12111	Collagen alpha-3(VI) chain	COL6A3	0.2770474	3.185E-09
P02751	Fibronectin	FN1	0.2716036	1.05E-05
O96005	Cleft lip and palate transmembrane protein 1	CLPTM1	0.2667236	5.165E-05
O15335	Chondroadherin	CHAD	0.2651015	0.0144708
P02724	Glycophorin-A	GYPA	0.2582686	0.0005169
Q15113	Procollagen C-endopeptidase enhancer 1	PCOLCE	0.255567	0.0001193
P51884	Lumican	LUM	0.2426271	5.734E-06
Q8NC56	LEM domain-containing protein 2	LEMD2	0.2366722	5.092E-05
Q96MM6	Heat shock 70 kDa protein 12B	HSPA12B	0.2333751	0.0020881
Q9Y6C2	EMILIN-1	EMILIN1	0.2328991	2.755E-06
P02452	Collagen alpha-1(I) chain	COL1A1	0.2251064	0.0002358
P35580	Myosin-10	MYH10	0.2231436	1.248E-05
Q14315	Filamin-C	FLNC	0.2192969	0.0043418
P05997	Collagen alpha-2(V) chain	COL5A2	0.2071277	1.582E-05
Q9NQR4	Omega-amidase NIT2	NIT2	0.1960614	0.0284942
P28300	Protein-lysine 6-oxidase	LOX	0.1901023	0.0006527
P08123	Collagen alpha-2(I) chain	COL1A2	0.1879446	1.47E-05
P20774	Mimecan	OGN	0.1864398	6.305E-06

Q6YHK3	CD109 antigen	CD109	0.1839302	0.0041837
P46821	Microtubule-associated protein 1B	MAP1B	0.1764101	6.147E-05
Q9ULL5	Proline-rich protein 12	PRR12	0.1756289	0.0002258
P20908	Collagen alpha-1(V) chain	COL5A1	0.1743121	5.238E-06
Q9H2X9	Solute carrier family 12 member 5	SLC12A5	0.173789	0.0004433
P02461	Collagen alpha-1(III) chain	COL3A1	0.1722658	9.057E-06
Q6UB35	Monofunctional C1-tetrahydrofolate synthase, mitochondrial	MTHFD1L	0.149144	0.004454
Q13425	Beta-2-syntrophin	SNTB2	0.1437103	0.0005336
P07305	Histone H1.0	H1-0	0.141131	1.348E-05
Q9BXN1	Asporin	ASPN	0.1403164	1.899E-06
Q99983	Osteomodulin	OMD	0.1327914	2.389E-06
P05186	Alkaline phosphatase, tissue-nonspecific isozyme	ALPL	0.1281213	6.72E-06
Q9NRN5	Olfactomedin-like protein 3	OLFML3	0.1130259	1.954E-06
P10915	Hyaluronan and proteoglycan link protein 1	HAPLN1	0.0525557	0.034089
P02689	Myelin P2 protein	PMP2	0.0452937	0.0330406
P24821	Tenascin	TNC	0.0353809	2.51E-11
P25189	Myelin protein P0	MPZ	0.0151504	0.0150574
Q9UQP3	Tenascin-N	TNN	0.0006232	0.0002005

Proteins marked red background: up-regulation in human apical periodontitis tissue compared with human periodontal ligament;
Proteins marked green background: down-regulation in human apical periodontitis tissue compared with human periodontal ligament.

Table S2. Proteins that consistent presence/ absence expression profile.

Protein	Protein Name	Gene Name
A0A075B6J9	Immunoglobulin lambda variable 2-18	IGLV2-18
A0A075B6K0	Immunoglobulin lambda variable 3-16	IGLV3-16
A0A075B6K5	Immunoglobulin lambda variable 3-9	IGLV3-9
A0A075B6P5	Immunoglobulin kappa variable 2-28	IGKV2-28
A0A0C4DH68	Immunoglobulin kappa variable 2-24	IGKV2-24
A0A087WSX0	Immunoglobulin lambda variable 5-45	IGLV5-45
A0A0B4J1U7	Immunoglobulin heavy variable 6-1	IGHV6-1
A0A0C4DH25	Immunoglobulin kappa variable 3D-20	IGKV3D-20
A0A0C4DH34	Immunoglobulin heavy variable 4-28	IGHV4-28
P0DP08	Immunoglobulin heavy variable 4-38-2	IGHV4-38-2
A0A0C4DH72	Immunoglobulin kappa variable 1-6	IGKV1-6
A0AVT1	Ubiquitin-like modifier-activating enzyme 6	UBA6
A8K2U0	Alpha-2-macroglobulin-like protein 1	A2ML1
B0I1T2	Unconventional myosin-Ig	MYO1G
G2XKQ0	Small ubiquitin-related modifier 5	SUMO1P1
O00154	Cytosolic acyl coenzyme A thioester hydrolase	ACOT7
O00204	Sulfotransferase 2B1	SULT2B1
O00217	NADH dehydrogenase [ubiquinone] iron-sulfur protein 8, mitochondrial	NDUFS8
O00244	Copper transport protein ATOX1	ATOX1
O00429	Dynamin-1-like protein	DNM1L
O00505	Importin subunit alpha-4	KPNA3
O00754	Lysosomal alpha-mannosidase	MAN2B1
O14745	Na(+)/H(+) exchange regulatory cofactor NHE-RF1	SLC9A3R1
O14949	Cytochrome b-c1 complex subunit 8	UQCRQ
O14980	Exportin-1	XPO1
O15347	High mobility group protein B3	HMGB3
O15371	Eukaryotic translation initiation factor 3 subunit D	EIF3D
O15533	Tapasin	TAPBP
O43252	Bifunctional 3-phosphoadenosine 5-phosphosulfate synthase 1	PAPSS1
O43598	2-deoxynucleoside 5-phosphate N-hydrolase 1	DNPH1
O43676	NADH dehydrogenase [ubiquinone] 1 beta subcomplex subunit 3	NDUFB3
O43760	Synaptogyrin-2	SYNGR2
O43813	Glutathione S-transferase LANCL1	LANCL1
O43837	Isocitrate dehydrogenase [NAD] subunit beta, mitochondrial	IDH3B
O60218	Aldo-keto reductase family 1 member B10	AKR1B10
O60725	Protein-S-isoprenylcysteine O-methyltransferase	ICMT
O75347	Tubulin-specific chaperone A	TBCA
O75438	NADH dehydrogenase [ubiquinone] 1 beta subcomplex subunit 1	NDUFB1
O75608	Acyl-protein thioesterase 1	LYPLA1
O75822	Eukaryotic translation initiation factor 3 subunit J	EIF3J

O75828	Carbonyl reductase [NADPH] 3	CBR3
O94826	Mitochondrial import receptor subunit TOM70	TOMM70
O94925	Glutaminase kidney isoform, mitochondrial	GLS
O95249	Golgi SNAP receptor complex member 1	GOSR1
O95299	NADH dehydrogenase [ubiquinone] 1 alpha subcomplex subunit 10, mitochondrial	NDUFA10
O95336	6-phosphogluconolactonase	PGLS
O95379	Tumor necrosis factor alpha-induced protein 8	TNFAIP8
O95433	Activator of 90 kDa heat shock protein ATPase homolog 1	AHSA1
O95470	Sphingosine-1-phosphate lyase 1	SGPL1
O95674	Phosphatidate cytidylyltransferase 2	CDS2
O95816	BAG family molecular chaperone regulator 2	BAG2
P00491	Purine nucleoside phosphorylase	PNP
P00492	Hypoxanthine-guanine phosphoribosyltransferase	HPRT1
P00966	Argininosuccinate synthase	ASS1
P01040	Cystatin-A	CSTA
P01624	Immunoglobulin kappa variable 3-15	IGKV3-15
P01700	Immunoglobulin lambda variable 1-47	IGLV1-47
P01701	Immunoglobulin lambda variable 1-51	IGLV1-51
P0DP03	Immunoglobulin heavy variable 3-30-5	IGHV3-30-5
P02760	Protein AMBP	AMBP
P02763	Alpha-1-acid glycoprotein 1	ORM1
P02786	Transferrin receptor protein 1	TFRC
P04233	HLA class II histocompatibility antigen gamma chain	CD74
P04259	Keratin, type II cytoskeletal 6B	KRT6B
P04430	Immunoglobulin kappa variable 1-16	IGKV1-16
P05198	Eukaryotic translation initiation factor 2 subunit 1	EIF2S1
P05783	Keratin, type I cytoskeletal 18	KRT18
P06310	Immunoglobulin kappa variable 2-30	IGKV2-30
P06312	Immunoglobulin kappa variable 4-1	IGKV4-1
P06454	Prothymosin alpha	PTMA
P07108	Acyl-CoA-binding protein	DBI
P07476	Involucrin	IVL
P07602	Prosaposin	PSAP
P07711	Cathepsin L1	CTSL
P08237	ATP-dependent 6-phosphofructokinase, muscle type	PFKM
P08240	Signal recognition particle receptor subunit alpha	SRPRA
P09104	Gamma-enolase	ENO2
P09467	Fructose-1,6-bisphosphatase 1	FBP1
P09497	Clathrin light chain B	CLTB
P09661	U2 small nuclear ribonucleoprotein A	SNRPA1
P09668	Pro-cathepsin H	CTSH
P09669	Cytochrome c oxidase subunit 6C	COX6C

P0DOX6	Immunoglobulin mu heavy chain	
P10153	Non-secretory ribonuclease	RNASE2
P10619	Lysosomal protective protein	CTSA
P10768	S-formylglutathione hydrolase	ESD
P11169	Solute carrier family 2, facilitated glucose transporter member 3	SLC2A3
P11182	Lipoamide acyltransferase component of branched-chain alpha-keto acid dehydrogenase complex, mitochondrial	DBT
P11234	Ras-related protein Ral-B	RALB
P11387	DNA topoisomerase 1	TOP1
P12004	Proliferating cell nuclear antigen	PCNA
P12724	Eosinophil cationic protein	RNASE3
P12830	Cadherin-1	CDH1
P13498	Cytochrome b-245 light chain	CYBA
P14317	Hematopoietic lineage cell-specific protein	HCLS1
P14324	Farnesyl pyrophosphate synthase	FDPS
P14923	Junction plakoglobin	JUP
P14927	Cytochrome b-c1 complex subunit 7	UQCRB
P15289	Arylsulfatase A	ARSA
P15531	Nucleoside diphosphate kinase A	NME1
P15927	Replication protein A 32 kDa subunit	RPA2
P15954	Cytochrome c oxidase subunit 7C, mitochondrial	COX7C
P16885	1-phosphatidylinositol 4,5-bisphosphate phosphodiesterase gamma-2	PLCG2
P17174	Aspartate aminotransferase, cytoplasmic	GOT1
P17900	Ganglioside GM2 activator	GM2A
P19623	Spermidine synthase	SRM
P20160	Azurocidin	AZU1
P20339	Ras-related protein Rab-5A	RAB5A
P20591	Interferon-induced GTP-binding protein Mx1	MX1
P20645	Cation-dependent mannose-6-phosphate receptor	M6PR
P20933	N(4)-(beta-N-acetylglucosaminyl)-L-asparaginase	AGA
P20962	Parathymosin	PTMS
P21399	Cytoplasmic aconitate hydratase	ACO1
P23588	Eukaryotic translation initiation factor 4B	EIF4B
P23786	Carnitine O-palmitoyltransferase 2, mitochondrial	CPT2
P33947	ER lumen protein-retaining receptor 2	KDELR2
P24557	Thromboxane-A synthase	TBXAS1
P25774	Cathepsin S	CTSS
P27482	Calmodulin-like protein 3	CALML3
P28161	Glutathione S-transferase Mu 2	GSTM2
P28907	ADP-ribosyl cyclase/cyclic ADP-ribose hydrolase 1	CD38
P29034	Protein S100-A2	S100A2
P29144	Tripeptidyl-peptidase 2	TPP2
P29466	Caspase-1	CASP1

P30405	Peptidyl-prolyl cis-trans isomerase F, mitochondrial	PPIF
P30419	Glycylpeptide N-tetradecanoyltransferase 1	NMT1
P31153	S-adenosylmethionine synthase isoform type-2	MAT2A
P31994	Low affinity immunoglobulin gamma Fc region receptor II-b	FCGR2B
P32320	Cytidine deaminase	CDA
P32926	Desmoglein-3	DSG3
P32942	Intercellular adhesion molecule 3	ICAM3
P33241	Lymphocyte-specific protein 1	LSP1
P35754	Glutaredoxin-1	GLRX
P35914	Hydroxymethylglutaryl-CoA lyase, mitochondrial	HMGCL
P36543	V-type proton ATPase subunit E 1	ATP6V1E1
P37235	Hippocalcin-like protein 1	HPCAL1
P40261	Nicotinamide N-methyltransferase	NNMT
P40763	Signal transducer and activator of transcription 3	STAT3
P41227	N-alpha-acetyltransferase 10	NAA10
Q04828	Aldo-keto reductase family 1 member C1	AKR1C1
P43007	Neutral amino acid transporter A	SLC1A4
P43304	Glycerol-3-phosphate dehydrogenase, mitochondrial	GPD2
P43686	26S proteasome regulatory subunit 6B	PSMC4
P46108	Adapter molecule crk	CRK
P46109	Crk-like protein	CRKL
P48449	Lanosterol synthase	LSS
P48506	Glutamate--cysteine ligase catalytic subunit	GCLC
P48723	Heat shock 70 kDa protein 13	HSPA13
P49327	Fatty acid synthase	FASN
P49720	Proteasome subunit beta type-3	PSMB3
P49773	Histidine triad nucleotide-binding protein 1	HINT1
P49863	Granzyme K	GZMK
P49913	Cathelicidin antimicrobial peptide	CAMP
P49961	Ectonucleoside triphosphate diphosphohydrolase 1	ENTPD1
P50135	Histamine N-methyltransferase	HNMT
P50579	Methionine aminopeptidase 2	METAP2
P51159	Ras-related protein Rab-27A	RAB27A
P51452	Dual specificity protein phosphatase 3	DUSP3
P51570	Galactokinase	GALK1
P51665	26S proteasome non-ATPase regulatory subunit 7	PSMD7
P51812	Ribosomal protein S6 kinase alpha-3	RPS6KA3
P51970	NADH dehydrogenase [ubiquinone] 1 alpha subcomplex subunit 8	NDUFA8
P52566	Rho GDP-dissociation inhibitor 2	ARHGDIB
P53634	Dipeptidyl peptidase 1	CTSC
P53992	Protein transport protein Sec24C	SEC24C
P54289	Voltage-dependent calcium channel subunit alpha-2/delta-1	CACNA2D1
P54821	Paired mesoderm homeobox protein 1	PRRX1

P55039	Developmentally-regulated GTP-binding protein 2	DRG2
Q5VTU8	ATP synthase subunit epsilon-like protein, mitochondrial	ATP5F1EP2
P57105	Synaptojanin-2-binding protein	SYNJ2BP
P57737	Coronin-7	CORO7
P58546	Myotrophin	MTPN
P61165	Transmembrane protein 258	TMEM258
P61513	60S ribosomal protein L37a	RPL37A
P61758	Prefoldin subunit 3	VBP1
P61964	WD repeat-containing protein 5	WDR5
P61970	Nuclear transport factor 2	NUTF2
P62191	26S proteasome regulatory subunit 4	PSMC1
P62273	40S ribosomal protein S29	RPS29
P62328	Thymosin beta-4	TMSB4X
P67775	Serine/threonine-protein phosphatase 2A catalytic subunit alpha isoform	PPP2CA
P62760	Visinin-like protein 1	VSNL1
P62995	Transformer-2 protein homolog beta	TRA2B
P67812	Signal peptidase complex catalytic subunit SEC11A	SEC11A
P78344	Eukaryotic translation initiation factor 4 gamma 2	EIF4G2
P79483	HLA class II histocompatibility antigen, DR beta 3 chain	HLA-DRB3
P80188	Neutrophil gelatinase-associated lipocalin	LCN2
P84085	ADP-ribosylation factor 5	ARF5
P84090	Enhancer of rudimentary homolog	ERH
Q01469	Fatty acid-binding protein 5	FABP5
Q01546	Keratin, type II cytoskeletal 2 oral	KRT76
Q02413	Desmoglein-1	DSG1
Q02790	Peptidyl-prolyl cis-trans isomerase FKBP4	FKBP4
Q03001	Dystonin	DST
Q04446	1,4-alpha-glucan-branching enzyme	GBE1
Q04760	Lactoylglutathione lyase	GLO1
Q07812	Apoptosis regulator BAX	BAX
Q08188	Protein-glutamine gamma-glutamyltransferase E	TGM3
Q10589	Bone marrow stromal antigen 2	BST2
Q12874	Splicing factor 3A subunit 3	SF3A3
Q13043	Serine/threonine-protein kinase 4	STK4
Q13148	TAR DNA-binding protein 43	TARDBP
Q13177	Serine/threonine-protein kinase PAK 2	PAK2
Q13185	Chromobox protein homolog 3	CBX3
Q13232	Nucleoside diphosphate kinase 3	NME3
Q13247	Serine/arginine-rich splicing factor 6	SRSF6
Q13526	Peptidyl-prolyl cis-trans isomerase NIMA-interacting 1	PIN1
Q13751	Laminin subunit beta-3	LAMB3
Q13753	Laminin subunit gamma-2	LAMC2
Q14005	Pro-interleukin-16	IL16

Q14161	ARF GTPase-activating protein GIT2	GIT2
Q14166	Tubulin--tyrosine ligase-like protein 12	TTLL12
Q14690	Protein RRP5 homolog	PDCD11
Q14696	LRP chaperone MESD	MESD
Q15043	Zinc transporter ZIP14	SLC39A14
Q15046	Lysine--tRNA ligase	KARS1
Q15118	[Pyruvate dehydrogenase (acetyl-transferring)] kinase isozyme 1, mitochondrial	PDK1
Q15125	3-beta-hydroxysteroid-Delta(8),Delta(7)-isomerase	EBP
Q15257	Serine/threonine-protein phosphatase 2A activator	PTPA
Q15306	Interferon regulatory factor 4	IRF4
Q15369	Elongin-C	ELOC
Q15437	Protein transport protein Sec23B	SEC23B
Q15819	Ubiquitin-conjugating enzyme E2 variant 2	UBE2V2
Q16706	Alpha-mannosidase 2	MAN2A1
Q16719	Kynureninase	KYNU
Q16775	Hydroxyacylglutathione hydrolase, mitochondrial	HAGH
Q16822	Phosphoenolpyruvate carboxykinase [GTP], mitochondrial	PCK2
Q2TAA2	Isoamyl acetate-hydrolyzing esterase 1 homolog	IAH1
Q4G0X9	Coiled-coil domain-containing protein 40	CCDC40
Q53EP0	Fibronectin type III domain-containing protein 3B	FNDC3B
Q5JRX3	Presequence protease, mitochondrial	PITRM1
Q5TZA2	Rootletin	CROCC
Q5VT79	Annexin A8-like protein 1	ANXA8L1
Q6IBS0	Twinfilin-2	TWF2
Q6NUQ4	Transmembrane protein 214	TMEM214
Q6P4A8	Phospholipase B-like 1	PLBD1
Q6PKG0	La-related protein 1	LARP1
Q6PL18	ATPase family AAA domain-containing protein 2	ATAD2
Q6UW02	Cytochrome P450 20A1	CYP20A1
Q6UWP8	Suprabasin	SBSN
Q6UX06	Olfactomedin-4	OLFM4
Q6UXH1	Protein disulfide isomerase CRELD2	CRELD2
Q6ZN66	Guanylate-binding protein 6	GBP6
Q709C8	Vacuolar protein sorting-associated protein 13C	VPS13C
Q7Z4W1	L-xylulose reductase	DCXR
Q86TM6	E3 ubiquitin-protein ligase synoviolin	SYVN1
Q86X76	Deaminated glutathione amidase	NIT1
Q8IXM6	Nurim	NRM
Q8IYB3	Serine/arginine repetitive matrix protein 1	SRRM1
Q8IZ83	Aldehyde dehydrogenase family 16 member A1	ALDH16A1
Q8N766	ER membrane protein complex subunit 1	EMC1
Q8NCA5	Protein FAM98A	FAM98A

Q8NEW0	Zinc transporter 7	SLC30A7
Q8NFV4	Protein ABHD11	ABHD11
Q8TCG1	Protein CIP2A	CIP2A
Q8TDY2	RB1-inducible coiled-coil protein 1	RB1CC1
Q8WUM0	Nuclear pore complex protein Nup133	NUP133
Q8WXA3	RUN and FYVE domain-containing protein 2	RUFY2
Q92608	Dedicator of cytokinesis protein 2	DOCK2
Q92882	Osteoclast-stimulating factor 1	OSTF1
Q93084	Sarcoplasmic/endoplasmic reticulum calcium ATPase 3	ATP2A3
Q9H2M3	S-methylmethionine--homocysteine S-methyltransferase BHMT2	BHMT2
Q969M3	Protein YIPF5	YIPF5
Q969Q0	60S ribosomal protein L36a-like	RPL36AL
Q969V3	Nicalin	NCLN
Q96AZ6	Interferon-stimulated gene 20 kDa protein	ISG20
Q96C86	m7GpppX diphosphatase	DCPS
Q96CN7	Isochorismatase domain-containing protein 1	ISOC1
Q96DI7	U5 small nuclear ribonucleoprotein 40 kDa protein	SNRNP40
Q96EY1	DnaJ homolog subfamily A member 3, mitochondrial	DNAJA3
Q96HE7	ERO1-like protein alpha	ERO1A
Q96IU4	Protein ABHD14B	ABHD14B
Q96KA5	Cleft lip and palate transmembrane protein 1-like protein	CLPTM1L
Q96M27	Protein PRRC1	PRRC1
Q96RQ1	Endoplasmic reticulum-Golgi intermediate compartment protein 2	ERGIC2
Q99729	Heterogeneous nuclear ribonucleoprotein A/B	HNRNPAB
Q9BQA9	Cytochrome b-245 chaperone 1	CYBC1
Q9BQE5	Apolipoprotein L2	APOL2
Q9BRX8	Peroxiredoxin-like 2A	PRXL2A
Q9BT22	Chitobiosyldiphosphodolichol beta-mannosyltransferase	ALG1
Q9BUN8	Derlin-1	DERL1
Q9BV40	Vesicle-associated membrane protein 8	VAMP8
Q9BW30	Tubulin polymerization-promoting protein family member 3	TPPP3
Q9BXJ9	N-alpha-acetyltransferase 15, NatA auxiliary subunit	NAA15
Q9BXS5	AP-1 complex subunit mu-1	AP1M1
Q9GZT3	SRA stem-loop-interacting RNA-binding protein, mitochondrial	SLIRP
Q9H013	Disintegrin and metalloproteinase domain-containing protein 19	ADAM19
Q9H0R4	Haloacid dehalogenase-like hydrolase domain-containing protein 2	HDHD2
Q9H2U2	Inorganic pyrophosphatase 2, mitochondrial	PPA2
Q9H444	Charged multivesicular body protein 4b	CHMP4B
Q9H488	GDP-fucose protein O-fucosyltransferase 1	POFUT1
Q9H553	Alpha-1,3/1,6-mannosyltransferase ALG2	ALG2
Q9HA77	Probable cysteine--tRNA ligase, mitochondrial	CARS2
Q9HAK2	Transcription factor COE2	EBF2
Q9HC07	Transmembrane protein 165	TMEM165

Q9HC38	Glyoxalase domain-containing protein 4	GLOD4
Q9HCN8	Stromal cell-derived factor 2-like protein 1	SDF2L1
Q9HCY8	Protein S100-A14	S100A14
Q9HD45	Transmembrane 9 superfamily member 3	TM9SF3
Q9NP97	Dynein light chain roadblock-type 1	DYNLRB1
Q9NR50	Translation initiation factor eIF-2B subunit gamma	EIF2B3
Q9NRV9	Heme-binding protein 1	HEBP1
Q9NUQ9	Protein FAM49B	FAM49B
Q9NX63	MICOS complex subunit MIC19	CHCHD3
Q9NY33	Dipeptidyl peptidase 3	DPP3
Q9P0J0	NADH dehydrogenase [ubiquinone] 1 alpha subcomplex subunit 13	NDUFA13
Q9P287	BRCA2 and CDKN1A-interacting protein	BCCIP
Q9P2J5	Leucine--tRNA ligase, cytoplasmic	LARS
Q9P2R7	Succinate--CoA ligase [ADP-forming] subunit beta, mitochondrial	SUCLA2
Q9UBC9	Small proline-rich protein 3	SPRR3
Q9UBG3	Cornulin	CRNN
Q9UBQ0	Vacuolar protein sorting-associated protein 29	VPS29
Q9UBV2	Protein sel-1 homolog 1	SEL1L
Q9UGI8	Testin	TES
Q9UHB9	Signal recognition particle subunit SRP68	SRP68
Q9UHL4	Dipeptidyl peptidase 2	DPP7
Q9UIV8	Serpin B13	SERPINB13
Q9UKK3	Protein mono-ADP-ribosyltransferase PARP4	PARP4
Q9UKK9	ADP-sugar pyrophosphatase	NUDT5
Q9UKV3	Apoptotic chromatin condensation inducer in the nucleus	ACIN1
Q9UKV8	Protein argonaute-2	AGO2
Q9ULC4	Malignant T-cell-amplified sequence 1	MCTS1
Q9ULZ3	Apoptosis-associated speck-like protein containing a CARD	PYCARD
Q9UMX0	Ubiquilin-1	UBQLN1
Q9UNZ2	NSFL1 cofactor p47	NSFL1C
Q9Y266	Nuclear migration protein nudC	NUDC
Q9Y274	Type 2 lactosamine alpha-2,3-sialyltransferase	ST3GAL6
Q9Y2V2	Calcium-regulated heat-stable protein 1	CARHSP1
Q9Y320	Thioredoxin-related transmembrane protein 2	TMX2
Q9Y3A6	Transmembrane emp24 domain-containing protein 5	TMED5
Q9Y3C8	Ubiquitin-fold modifier-conjugating enzyme 1	UFC1
Q9Y4Z0	U6 snRNA-associated Sm-like protein LSm4	LSM4
Q9Y5K5	Ubiquitin carboxyl-terminal hydrolase isozyme L5	UCHL5
Q9Y5P6	Mannose-1-phosphate guanyltransferase beta	GMPPB
Q9Y6B6	GTP-binding protein SAR1b	SAR1B
Q9Y6E0	Serine/threonine-protein kinase 24	STK24
O00339	Matrilin-2	MATN2
O14498	Immunoglobulin superfamily containing leucine-rich repeat protein	ISLR

O15061	Synemin	SYNM
O43592	Exportin-T	XPOT
O75487	Glypican-4	GPC4
P00441	Superoxide dismutase [Cu-Zn]	SOD1
P02686	Myelin basic protein	MBP
P07197	Neurofilament medium polypeptide	NEFM
P08493	Matrix Gla protein	MGP
P09471	Guanine nucleotide-binding protein G(o) subunit alpha	GNAO1
P22413	Ectonucleotide pyrophosphatase/phosphodiesterase family member 1	ENPP1
P49746	Thrombospondin-3	THBS3
P50281	Matrix metalloproteinase-14	MMP14
P78539	Sushi repeat-containing protein SRPX	SRPX
P78559	Microtubule-associated protein 1A	MAP1A
Q00796	Sorbitol dehydrogenase	SORD
Q05193	Dynamin-1	DNM1
Q05397	Focal adhesion kinase 1	PTK2
Q15154	Pericentriolar material 1 protein	PCM1
Q63HR2	Tensin-2	TNS2
Q6UVK1	Chondroitin sulfate proteoglycan 4	CSPG4
Q8NCW0	Kremen protein 2	KREMEN2
Q8WXQ8	Carboxypeptidase A5	CPA5
Q96JN2	Coiled-coil domain-containing protein 136	CCDC136
Q96MA6	Adenylate kinase 8	AK8
Q96P44	Collagen alpha-1(XXI) chain	COL21A1
Q9BXM0	Periaxin	PRX
Q9BXX0	EMILIN-2	EMILIN2
Q9H1E3	Nuclear ubiquitous casein and cyclin-dependent kinase substrate 1	NUCKS1
Q9H8L6	Multimerin-2	MMRN2
Q9NUP9	Protein lin-7 homolog C	LIN7C
Q9NZW5	MAGUK p55 subfamily member 6	MPP6
Q9UPW6	DNA-binding protein SATB2	SATB2
Q9Y240	C-type lectin domain family 11 member A	CLEC11A
Q9Y2D5	A-kinase anchor protein 2	AKAP2
Q9Y2J2	Band 4.1-like protein 3	EPB41L3
Q9Y4G6	Talin-2	TLN2

Proteins in red font: detected in human apical periodontitis tissue but not in human periodontal ligament;

Proteins in green font: detected in human periodontal ligament but not in human apical periodontitis tissue.
